# Predicting Individual Preferences in Mindfulness Techniques Using Personality Traits

**DOI:** 10.3389/fpsyg.2020.01163

**Published:** 2020-06-18

**Authors:** Rongxiang Tang, Todd S. Braver

**Affiliations:** Department of Psychological and Brain Sciences, Washington University, St. Louis, MO, United States

**Keywords:** mindfulness, meditation, individual difference, personality, disposition, preferences

## Abstract

The growing popularity of mindfulness-based interventions (MBIs) has prompted exciting scientific research investigating their beneficial effects on well-being and health. Most mindfulness programs are provided as multi-faceted packages encompassing a set of different mindfulness techniques, each with distinct focus and mechanisms. However, this approach overlooks potential individual differences, which may arise in response to practicing various mindfulness techniques. The present study investigated preferences for four prototypical mindfulness techniques [focused attention (FA), open monitoring (OM), loving-kindness (LK), and body scan (BS)] and identified factors that may contribute to individual differences in these preferences. Participants without prior mindfulness experiences were exposed to each technique through audio-guided instructions and were asked to rank their preferences at the end of all practices. Results indicated that preferences for loving-kindness were predicted by empathy, and that females tended to prefer loving-kindness more than males. Conversely, preferences for open monitoring were predicted by nonreactivity and nonjudgment of present moment experiences. Additionally, higher state mindfulness was detected for individuals’ preferred technique relative to other alternatives. These findings suggest that individuals tend to prefer techniques compatible with their personalities, as the predictor variables encompass trait capacities specifically relevant to practicing these techniques. Together, our results suggest the possibility that assessing individual difference and then tailoring MBIs to individual needs could be a useful way to improve intervention effectiveness and subsequent outcomes.

## Introduction

Mindfulness-based interventions (MBIs) have gained traction over the past few decades among scientific and public communities for their promising effects in improving psychological well-being, cognition, physiology, and brain health, in both healthy and clinical populations (Chiesa et al., 2011; Gu et al., 2015; Khoury et al., 2015; Tang et al., 2015; Black and Slavich, 2016). Cultivating and promoting mindfulness – the nonjudgmental and nonreactive attention and awareness of present moment experiences – is the one of the central themes of MBIs (Kabat-Zinn, 1990; Hölzel et al., 2011). Most MBIs, including two of the most popular variants – mindfulness-based stress reduction (MBSR) (Kabat-Zinn, 1990) and mindfulness-based cognitive behavioral therapy (MBCT) (Segal et al., 2002) – are provided as multi-faceted intervention packages encompassing a variety of mindfulness techniques that together achieve the goals of intervention. While these different techniques share the same goal of promoting mindfulness, studies have increasingly suggested that distinct cognitive and emotional processes are engaged in these mindfulness practices (Lutz et al., 2008; Hölzel et al., 2011; Vago and Silbersweig, 2012).

Indeed, mindfulness techniques not only differ considerably in terms of their object of attentional focus, but also vary in how attentional focus is directed toward the observed object. Focused attention (FA), one of the common mindfulness techniques taught in MBIs, involves directing and maintaining attentional focus and awareness on a specific object or sensation (e.g., breathing) throughout the duration of practice (Lutz et al., 2008). In contrast, open monitoring (OM), another prototypical mindfulness technique, emphasizes a non-specific or non-deliberate attentional focus, which involves openly observing and accepting any sensation, thoughts, or emotion that arise at the present moment (Lutz et al., 2008). The former technique is concentrative and relies heavily on sustained attentional control to minimize mind wandering and distraction, while the latter technique is receptive and underscores a nonjudgmental monitoring of ongoing external and internal stimuli. Relatedly, loving-kindness, another common and popular mindfulness practice found in MBIs is vastly different from the previous two techniques. This practice involves cultivating positive emotions and feelings of kindness and love toward oneself and others through words and imagery, with the desire for all beings to be safe, happy, healthy, and free of suffering (Salzberg, 2011). This technique still requires individuals to be mindful during the practice, but with a main emphasis on fostering positive emotions and compassion toward oneself and others (Fredrickson et al., 2008; Hutcherson et al., 2008). These apparent differences in styles of mindfulness practice have inspired growing investigations delineating the mechanisms of action by which individual techniques work to differentially contribute to improvement in psychological and cognitive outcomes (Manna et al., 2010; Hölzel et al., 2011; Ainsworth et al., 2013; Lippelt et al., 2014; Britton et al., 2018). Most importantly, these investigations do not assume different mindfulness techniques would have homogenous effects on relevant outcomes, even though they are often taught together in standardized MBIs.

Following this line of logic, it is improbable to assume that each individual would show similar responsiveness to every mindfulness technique (Tang and Braver, 2020). In fact, recent evidence has already indicated that individuals have preferences toward specific meditation techniques. In a study of 247 undergraduate students who were exposed to four different types of meditation (Zen, Vipassana, Qigong, and Mantra) over the course of a 6-week training period, preferences were clearly demonstrated at the individual level, such that more students preferred Vipassana, a mindfulness-based technique that focuses on breath observation, or Mantra, a practice that involves mental repetition of a sound or Mantra, relative to the two other types of meditation – Qigong and Zen (Burke, 2012). Similarly, individual preferences or partiality toward specific modalities of attentional anchors (e.g., auditory, visual, and somatosensory) commonly used in mindfulness techniques have been demonstrated in individuals exposed to 10-min of meditative practices (Anderson and Farb, 2018). These findings together corroborate the notion that individual differences can be found in response to various mindfulness techniques, while also highlighting the importance of developing a better understanding of the potential moderating role of individual differences in MBI research – a need that has previously been underscored by a number of researchers in the field (Davidson, 2010; Farias and Wikholm, 2016). Indeed, as concerns for potential adverse effects of MBIs are increasingly being noted (Farias and Wikholm, 2016; Van Dam et al., 2018; Baer et al., 2019), attending to individual preferences and differences in interventions could have important implications for clinical applications. Furthermore, matching individuals with appropriate mindfulness techniques or other suitable alternative based on their preferences could reduce the likelihood of attrition, facilitate adherence, and self-maintenance of practices, thereby enhancing intervention effectiveness, and outcomes (Swift et al., 2011; Anderson and Farb, 2018).

However, there is currently a lack of knowledge of putative factors that may give rise to individual differences in preference, which makes it challenging to derive accurate predictions, and appropriately match individuals with techniques that they would likely find most useful. Based on prior studies of individual differences in psychotherapy and MBIs, personality and dispositional traits are promising candidates that have been shown to influence standardized intervention effectiveness and outcomes (Chapman et al., 2014; de Vibe et al., 2015; Nyklíček and Irrmischer, 2017). Furthermore, a recent study showed support for utilizing personality traits to predict individual preferences in mindfulness techniques, in that traits such as agreeableness and openness to experience, were predictive of the frequency of practicing mindfulness techniques (yoga, sitting meditation, informal meditation, and body scanning) during the period of MBSR intervention (Barkan et al., 2016). In particular, high openness predicted greater use of a variety of MBSR techniques both during and at 6-month follow-up of MBSR. The greater use of mindfulness techniques both during and after this standardized MBI may in fact suggest some extent of preferences were involved. Yet, no study has directly investigated the feasibility of using personality and dispositional traits as potential predictors of individual preferences for mindfulness techniques. The present study examined and identified relevant personality and dispositional traits that may predict individual preferences for four prototypical mindfulness techniques found in most MBIs: focused attention, open monitoring, loving-kindness, and body scan. Additionally, we explored whether preferences for specific mindfulness techniques would translate into differential level of state mindfulness in both bodily sensations and mental activities during each meditative practice, demonstrating the practical implications of understanding and predicting individual preferences in MBIs.

### Predictors of Individual Preferences for Mindfulness Techniques

To investigate whether personality and dispositional traits could be predictive of individual preferences, we specifically identified traits that encompass critical capacities and processing styles relevant for each mindfulness technique. The general hypothesis was that individuals would likely prefer techniques they can easily learn and practice without much difficulty or struggle. For example, a mindfulness technique that heavily engages attentional control is likely to be preferred by individuals who feel skilled in the engagement of attentional control. Based on this hypothesis, below we described candidate predictors for each mindfulness technique, as well as related hypotheses.

#### Open Monitoring

This mindfulness technique does not have a designated object of attentional focus; instead it engages meta-awareness of arising and passing stimuli and emphasizes an equanimous monitoring and acceptance of these present moment experiences (Vago and Silbersweig, 2012). Therefore, we hypothesized that two facets of trait mindfulness, *nonreactivity to inner experience* and *nonjudging of inner experience*, should predict more preference for OM. Additionally, we also hypothesized that *openness to experience*, a personality trait that involves open-mindedness, a sense of curiosity and attentiveness to inner feelings, should positively predict preferences for OM, as this trait should facilitate open awareness and acceptance of present moment experiences during the practice of OM.

#### Loving-Kindness

Loving-kindness (LK) is another commonly practiced technique in MBIs, which involves fostering loving-kindness toward oneself and others through mental imagery or words (Salzberg, 2011). The standard technique begins with cultivating kindness toward oneself, then progresses onto a friend or loved one, someone who is “difficult” or unkind (i.e., enemy or disliked person), and finally expanding it to all people (Hofmann et al., 2011). Given the emotional component of LK, we hypothesized that *empathy*, the ability to understand and adopt the psychological perspective of others and have feelings of sympathy for unfortunate others (Davis, 1983), should predict more preferences for LK. We also predicted that *self-compassion*, a dispositional trait that entails compassion and kindness toward oneself when encountering perceived failure and personal suffering (Neff, 2016), should predict more preferences for LK. Lastly, *agreeableness*, a personality trait characterized by similar qualities such as friendliness, warmth, and cooperativeness (McCrae and Costa, 1991) should also predict more preferences for LK.

#### Focused Attention

The recruitment of attentional control is necessary for reducing distraction and maintaining attentional focus in this technique (Lutz et al., 2008). Thus, we hypothesized that individual’s capacity of *attentional focusing*, the ability to maintain attention while inhibiting distraction should positively predict preferences for FA. Furthermore, *conscientiousness*, a personality trait characterized by industriousness, persistence, and self-control (Sanderson and Clarkin, 1994) should positively predict preferences for FA, as this trait has been shown to associate with inhibitory control and aspects of executive functions that support sustained attentional control (Vago and Silbersweig, 2012; Hall and Fong, 2013). Finally, *acting with awareness*, one facet of trait mindfulness that entails the ability to attend to present moment activities with awareness rather than behaving automatically, should predict preferences for FA.

#### Body Scan

Body scan is a technique that involves noticing and observing the physical sensation of individual body parts from top to bottom and sequentially shifting this attentional focus from one body part to the next (Dreeben et al., 2013). In particular, this technique has a cycle of directing, maintaining, disengaging, and shifting attention and awareness throughout the body. We hypothesized that *attentional shifting*, the ability to intentionally shift the attentional focus to desired object of focus, thereby avoiding unintentional focusing on other objects, should predict preferences for BS, since flexibly and constantly moving attentional focus is essential to this practice. Additionally, we hypothesized that *sensory processing sensitivity*, the tendency to engage in deeper cognitive processing of physical, emotional, and social stimuli or to show heightened response to such stimuli (Aron and Aron, 1997) should predict less preference for BS, as individuals with high sensory processing sensitivity would likely have a difficult time disengaging from the current sensory experience or become too overwhelmed by the experience.

### Covariates

Age and gender were included as standard covariates in all regression models. Additionally, we included the order in which participants practiced each technique as a covariate in all models to control any potential practice effects related to the randomization of practice order. As a supplemental exploratory analysis, we also examined the effects of controlling two other covariates – perceived stress and absorption. Perceived stress refers to individual’s recent perception of stress (Cohen and Williamson, 1988), which may have potential influence over preferences for mindfulness techniques, as mindfulness practices have generally been shown to reduce stress. Absorption describes the tendency to become fully engaged and devoted to sensory, imaginative, and self-altering experiences (perceptual, enactive, imaginative, and ideational) (Tellegen and Atkinson, 1974). It has previously been shown to influence depth of meditative experiences, regardless of which meditation technique or tradition was practiced by the individual (Hölzel and Ott, 2006). Consequently, varying meditation depth could potentially bias individual preferences for mindfulness techniques. Therefore, we did not include absorption as a primary predictor (instead as a covariate), given that its general effect on meditation depth is not specific to any particular technique. Results of the exploratory analysis with perceived stress and absorption as covariates are reported in the Supplementary Materials, but are also discussed briefly in the main text.

## Materials and Methods

### Participants

Participants without prior experiences in mindfulness and yoga practices were recruited for the study *via* the Amazon Mechanical Turk (MTurk) online platform. The TurkPrime interface was used to post study descriptions to MTurk, manage recruitment and payment, send out reminder emails, and handle all other communication with the participants. After reading a description of this multi-session study on MTurk, interested participants accessed a link which contained the consent form for them to review and sign electronically. After signing the consent, the web link for the pre-screening questions was made available over MTurk. Data were collected across two separate testing waves held a few months apart, but the procedures for the tasks were identical across waves. Combining data from both waves, 200 participants were initially recruited for this multi-session study. A total of 125 participants (79 males and 46 females) completed all study sessions, with ages ranging from 22 to 67 years old (*M* = 36.9, *SD* = 10.0). The drop-out participants were on average younger than those who completed all sessions. They also had higher extraversion, but lower agreeableness, openness to experience, and conscientiousness than participants who completed the study (see Supplementary Materials for more details). Participants were compensated a total of $16 for completing all sessions. All protocols were approved by the Washington University Institutional Review Board.

### Study Design and Procedure

The study included five sessions. Participants were informed that the study involved practicing mindfulness techniques through audio-guided recordings, answering short questions about the practices, and completing questionnaires. They were asked to complete one session per day for 5 consecutive days. On each day, a new session was posted and participants were notified *via* emails. A brief screening questionnaire of prior mindfulness and yoga experiences occurred before the first session of the experiment. Eligible participants with no prior practice experiences were then presented with the web link to the first session of the experiment. This first session included a set of self-report questionnaires tapping into individual differences in personality and dispositional traits, as well as basic demographic information. To ensure all mindfulness-naïve participants had a basic understanding of mindfulness practices before the subsequent audio-guided practice sessions (session 2–5), the first session also included a brief section with simple written descriptions that introduced participants to each of the four mindfulness techniques (Focused Attention, Open Monitoring, Loving-kindness, and Body Scan), followed by four probe questions that asked participants to briefly describe the differences among these techniques. Given the online nature of this study, these questions also served as an initial quality control step to exclude participants who clearly did not devote any effort into reading instructions or answering questions (e.g., filling out random or arbitrary answers) from later sessions.

The first session took 45–50 min to complete, whereas sessions 2–5 each took 15–20 min to complete. In sessions 2–5, participants practiced one of the four mindfulness techniques for 10 min by following audio-only instructions prerecorded by a certified MBSR instructor. Before the start of the practice, participants were asked to find a quiet place and to practice without interruption. Otherwise, they were asked to find a different time to come back for the practice. The practice order of the four techniques (sessions 2–5) was completely randomized for each participant. After the practice, participants were asked to take notes about their practice experiences, submitted them online, and saved their notes to a local computer. They were also informed that they would be using these notes in the last session for preference ranking of the four mindfulness techniques. Lastly, at the end of each session (2–5), participants completed the state mindfulness scale (SMS), based on the mindfulness practice experience they just had. In session 5, after completing the practice and SMS, participants were asked to copy and paste their previous practice notes into the textbox, review theses notes, and then rank all four techniques from *most preferred* to *least preferred*.

### Materials

#### Mindfulness Practices

For each of the four mindfulness techniques (Focused Attention, Open Monitoring, Loving-kindness, and Body Scan), a MBSR instructor recorded a 10-min audio-guided practice with standard instructions. Participants were instructed to find a comfortable position to sit down and begin the practice. As a quality control step, participants were not able to move onto the subsequent portion of the session until the 10-min audio instruction ended.

#### Preferences Ranking

Participants were asked to carefully review their practice notes from all four sessions to determine the preferences ranking of all four mindfulness techniques from 1 *most preferred* to 4 *least preferred*. As a validation step, participants were also asked to provide reasons for their rankings.

#### State Mindfulness

The SMS is a 21-item self-report questionnaire designed to measure mindfulness as a state-like phenomenon, rather than a stable trait. Scores for each item can range from 1 (not at all) to 5 (very well). The scale has been validated to be a reliable instrument (Cronbach’s *α* = 0.85–0.97) for assessing the level of present moment attention and awareness during a specific period of time (e.g., 10 min) and context (e.g., mindfulness practice) (Tanay and Bernstein, 2013). There are two subscales within this scale, one for assessing the level of mindfulness on bodily sensations, and the other for mental activities. For analyses reported here, the total score across the two subscales was used, as it indexes general state mindfulness, and also has good validity and reliability (Tanay and Bernstein, 2013).

#### Trait Mindfulness

The five facet mindfulness questionnaire (FFMQ), a widely used questionnaire was utilized for assessing individual differences in trait mindfulness. The FFMQ contains 39 items that range from 1 (never or very rarely true) to 5 (very often or always true), measuring five different facets of mindfulness (non-judging, non-reactivity, observing, describing, and acting with awareness). The FFMQ has also been widely used in previous studies and has good internal consistency with a Cronbach’s alpha ranging from 0.80 to 0.91 (Baer et al., 2008).

#### Big Five Personality

The 60-item NEO-five factor inventory (NEO-FFI) is a well-validated, reliable, and extensively used instrument by numerous studies in different populations for assessing five dimensions of personality, including neuroticism, extraversion, conscientiousness, agreeableness, and openness to experience (Costa and McCrae, 1989). Score for each item can range from 1 (strongly disagree) to 5 (strongly agree). The inventory has been shown to have good reliability for all five dimensions (Cronbach’s *α* = 0.75–0.85) (Sherry et al., 2007).

#### Self-Compassion

The self-compassion scale (SCS) contains 26 items that measures one’s tendency to be accepting, understanding, and caring toward oneself when facing failures, struggles, and negative emotions in life (Neff, 2003). Score for each item can range from 1 (almost never) to 5 (almost always). The SCS has been shown to have high test-retest reliability across all of its six subscales (*r* > 0.80) (Neff, 2003). The use of a total score has also been found to be reliable and valid for tapping into self-compassion in five different populations (Neff, 2016).

#### Interpersonal Reactivity Index

The interpersonal reactivity index (IRI) is 28-items self-report measure of individual differences in empathy and is scored on a five-point Likert scale ranging from “Does not describe me well” to “Describes me very well” (Davis, 1983). The measure has four subscales tapping into different aspects of empathy: Perspective Taking, Fantasy, Empathic Concern, and Personal Distress. The IRI is a valid and reliable measure of empathic tendencies with Cronbach’s alpha coefficients ranging from 0.75 to 0.80 and test-retest reliability (ICC) ranging from 0.61 to 0.81 (Davis, 1983; Baldner and McGinley, 2014).

#### Perceived Stress Scale

The perceived stress scale (PSS) is a widely used measure for assessing individual’s recent perception of stress and the degree to which situations in one’s life are deemed stressful during the last month (Cohen et al., 1983). The scale has 10 items and each item is scored on a five-point Likert scale ranging from “never” to “very often.” The PSS also has good reliability with a Cronbach’s alpha of 0.78 (Cohen et al., 1983).

#### Highly Sensitive Person Scale

The highly sensitive person (HSP) scale is a 27-item measure of sensory processing sensitivity (Aron and Aron, 1997). Score of each item ranges from 1 (not at all) to 7 (extremely). The HSP has been shown to be a reliable measure of individual differences in sensory processing sensitivity in different populations with Cronbach’s alpha ranging from 0.85 to 0.87 across different samples (Benham, 2006).

#### Attentional Control Scale

The attentional control scale (ACS) is a widely used self-report measure for assessing individual differences in attentional control. The ACS includes 20 items and two subscales on attention focusing and attention shifting. Score of each item ranges from 1 (almost never) to 4 (always). The ACS has shown good internal consistency for the full scale (*α* = 0.84), as well as for the attention focusing subscale (*α* = 0.87) and the attention shifting subscale (*α* = 0.77) (Derryberry and Reed, 2002; Judah et al., 2014).

#### Tellegen Absorption Scale

The Tellegen absorption scale (TAS) is a self-report measure of absorption, which refers to an individual’s tendency to have their attention fully engaged and devoted to sensory and imaginative experiences (perceptual, enactive, imaginative, and ideational) (Tellegen and Atkinson, 1974). The TAS has 34 items and each item is scored on a dichotomous scale (true or false). The scale has shown good internal reliability (*r* = 0.88) and test-retest reliability (*r* = 0.85–0.91).

### Data Analysis

Additional quality control steps were taken before data analysis to ensure we only include participants who followed instructions and at least devoted effort to complete each session. Therefore, for each participant, the practice notes from all four sessions were reviewed and participants with irrelevant, short, or arbitrary responses (e.g., good, fine) in more than one session were excluded from subsequent analyses. A total of five participants were excluded for this reason.

Guided by our hypotheses, data analysis was conducted in two phases. In the first phase, we tested whether the predictor variables theoretically hypothesized to be relevant for a given technique would be able to explain variation in preference for that technique. This first phase was useful for identifying meaningful candidate predictor variables that were consistent with our theoretical hypotheses, thus providing an initial level of support. However, it is important to note that this first phase was *not* geared toward demonstrating specificity, i.e., whether candidate predictor variables would predict certain techniques but not others. Therefore, in the second phase, we examined dissociable preferences in predictors to test for such specificity, by including the additional candidate predictors identified in the first phase. This step enabled a formal test of the relative predictive power of each candidate variable compared to others for a given mindfulness technique of interest.

To assess whether the hypothesized individual difference variables could predict individual preference rankings for the four mindfulness techniques, ordinal logistic regressions were first conducted using the *ordinal* and *rms* packages from R, since the rankings (*most preferred* to *least preferred*) were scored on an ordinal scale. Within each model, theoretically hypothesized personality traits and characteristics were used as predictors for preference rankings of each technique. Age, gender, and practice order were included in these regression models as covariates. For each predictor, a positive coefficient suggests increased likelihood (odds ratio) of ranking the technique in the more preferred categories and *vice versa* for negative coefficients. It should be noted that one important assumption of ordinal logistic regression is the proportional odds (PO) assumption, which states that the predictors would have the same effects across different thresholds of the dependent variable (i.e., ranking levels). In other words, it assumes that the coefficients that describe the relationship between the least preferred category versus all other preferred categories are the same as those that describe the relationship between the third preferred category and all other higher preferred categories (i.e., second preferred and most preferred) and so on. Therefore, the coefficient of each predictor would be the same regardless of the threshold, but the intercept at each threshold would be different from one another. We specifically checked for this PO assumption through the Brant test (Brant, 1990) using the *Brant* package in R. This test showed that the PO assumption was violated by two different predictors (see below for details). Hence, multinomial logistic regressions (MNL) were also conducted as a follow-up analysis whenever the PO assumption was violated by the predictors, using the *multinom* package from R.

The logic behind MNL is different from ordinal logistic regression, in that MNL treats the dependent variable (i.e., rankings) as unordered but allows the coefficients of each predictor to differ across the thresholds, which partially mitigates the constraint imposed by the PO assumption. For MNL, a reference category is always set, to which all other categories (i.e., ranking levels) can be compared. For our MNL models, the reference category was set to “*most preferred*,” meaning that all other ranking levels (i.e., categories) would be compared relative to this base category. Specifically, the MNL model estimated three sets of binary logit models: (1) most preferred vs. second preferred, (2) most preferred vs. third preferred, and (3) most preferred vs. least preferred. Consequently, different estimates of coefficients for the predictors were obtained from the three models and positive coefficients entail an increase in the odds of ranking the technique in the present category (often the less preferred categories), compared to the reference category (i.e., most preferred), and *vice versa* for negative coefficients. Because MNL does not constrain the predictors to have the same coefficients across all levels of the dependent variable, three different sets of coefficients would be obtained for each of the three binary logistic regressions. In the following section, we presented results from both ordinal logistic regression and MNL to illustrate the relationship between individual differences in personality traits and preferences for mindfulness techniques.

## Results

### Preferences Distribution

**Figure 1:**
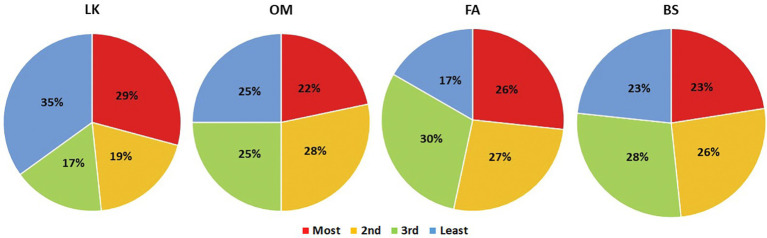
Preferences distribution of mindfulness techniques.

Preference rankings for the four mindfulness techniques were mostly evenly distributed as shown in Figure 1, indicating that we were able to capture a good range of variability with regard to individual preferences. Notably, participants did not exhibit systematic biases toward one technique or another. Interestingly, across the four techniques, loving-kindness was ranked as the most preferred technique by 29% of the participants but was also ranked as the least preferred technique by 35% of the participants. None of the other techniques, showed any bimodal trend.

### Loving-Kindness

Perspective taking, empathic concern, and fantasy subscales from the IRI were summed into a composite score to represent empathy, as this approach would eliminate multicollinearity among these subscales of IRI. Personal distress, a subscale of IRI was not included in this composite because it has shown a negative correlation with all other subscales and does not load onto the empathy factor, in investigations of the hierarchical factor structure of IRI (Pulos et al., 2004). In addition to empathy, self-compassion (the total score) was also included as a predictor in the model (Model 1). Although agreeableness was another predictor of interest, it was highly correlated with empathy (*r* = 0.44, *p* < 0.001). Thus, we ran a separate model (Model 2) with agreeableness and all the above variables except empathy. Table 1 shows the coefficients, standard errors, and significance level for each predictor in each model. The AIC of each model is also shown.

**Table 1:** Ordinal logistic regression of preferences for loving-kindness.

	Model 1		Model 2	
Predictors	*Coefficient*	*SE*	*Coefficient*	*SE*
Empathy	0.066^***^	0.015	-	-
Agreeableness	-	-	0.054^*^	0.027
Self-compassion	−0.199	0.203	−0.185	0.209
Age	−0.022	0.018	−0.017	0.018
Gender	0.921^*^	0.380	1.157^***^	0.371
Practice order	−0.331^*^	0.165	−0.256	0.158
Model fit AIC	292.77		308.33	

* Indicates *p* < 0.05;

** Indicates *p* < 0.01;

*** Indicates *p* < 0.005.

Results showed that in Model 1, empathy significantly predicted preferences for LK, such that an increase in empathy was associated with greater probability of ranking LK in more preferred categories. In other words, if we convert the coefficient of empathy to odds ratio, for one unit of increase in empathy, the odds of ranking LK as the most preferred technique versus all other categories increased by 6.8% (*e*^0.066^), while controlling for other variables. Additionally, gender also showed significance, such that females were more likely to rank LK as their most preferred technique than males, and that the odds of doing so were 151% (*e*^0.921^) higher than that of males. Practice order was another covariate that showed significance, suggesting that the later the individuals practiced LK, the less likely they were to rank LK as their most preferred technique. For Model 2, agreeableness was a significant predictor, such that higher agreeableness was associated with greater likelihood of ranking LK as the most preferred technique. Gender also showed significance in this model, with females again showing more preferences for LK than males. Comparing Model fit, Model 1 had a higher AIC than Model 2, suggesting a better fit of this model, which includes empathy as the primary predictor. Finally, we added agreeableness to Model 1 in order to see if there would be any changes in the predictive power of empathy. In this new combined model, the significance of agreeableness went away, but empathy and gender, as well as practice order, remained to be significant predictors even after controlling for the effect of agreeableness. An exploratory analysis that included perceived stress and absorption as additional covariates also corroborated the above findings by showing the same significant predictors.

Among all of the predictors, self-compassion was the only predictor that violated the PO assumption. Therefore, we also ran multinomial logistic regressions using predictors from Model 1 as follow-up to ordinal logistic regressions. Not surprisingly, as shown in Table 2, empathy still positively and significantly predicted preferences for LK across all categories (i.e., second preferred, third preferred, and least preferred), such that individuals with higher empathy were more likely to rank LK as their most preferred technique above all other techniques. Surprisingly, self-compassion showed significance in MNL models, but in the opposite direction, as it *negatively* predicted preferences for LK. Yet, it was only significant as a predictor for two categories (i.e., second preferred, third preferred), so the result should be treated with caution. The pattern of findings suggested that people who scored high on self-compassion were more likely to rank LK as their second preferred technique rather than the most preferred, or rank it as the third preferred technique, rather than the most preferred. For covariates, gender and practice order showed significant predictability in the least preferred category, such that females were more likely to prefer LK than males and rank it as the most preferred technique, rather than the least preferred, and that the later the individuals practiced LK, the more likely they were to rank it as the least preferred technique, rather than the most preferred.

**Table 2:** Multinomial logistic regression of preferences for loving-kindness.

	Second preferred		Third preferred		Least preferred	
Predictors	*Coefficient*	*SE*	*Coefficient*	*SE*	*Coefficient*	*SE*
Empathy	−0.095^***^	0.030	−0.109^***^	0.031	−0.136^***^	0.030
Self-compassion	0.963^**^	0.346	1.070^***^	0.377	0.488	0.330
Age	−0.024	0.031	−0.007	0.032	0.022	0.027
Gender	−0.934	0.611	−0.922	0.671	−1.839^***^	0.613
Practice order	0.073	0.274	−0.194	0.312	0.620^*^	0.269

* Indicates *p* < 0.05;

** Indicates *p* < 0.01;

*** Indicates *p* < 0.005.

### Open Monitoring

Two facets of trait mindfulness from the FFMQ – non-judging of inner experiences and non-reactivity to inner experiences were included as predictors in the ordinal logistic regression model. In particular, the two facets were summed into one composite to eliminate multicollinearity among the predictors. The observing facet was not included in this composite as prior literature has shown that this facet does not correlate well with other facets or load onto the overall mindfulness trait in non-meditating samples and may in fact represent neutral attention, rather than mindful observing (Gu et al., 2016). Openness to experience was also included in the model as another predictor. However, the trait mindfulness composite was the only significant predictor as shown in Table 3. Specifically, for one unit of increase in mindfulness composite, the odds of ranking OM as the most preferred technique increased by 43% (*e*^0.356^). Furthermore, in an exploratory model including perceived stress and absorption as covariates, mindfulness composite was still a positive predictor that showed significance, but perceived stress was found to be a significant covariate, such that individuals with higher levels of perceived stress were more likely to rank OM as the most preferred technique.

**Table 3:** Ordinal logistic regression of preferences for open monitoring.

Predictors	Coefficient	SE
Mindfulness composite	0.356^**^	0.130
Openness to experience	−0.007	0.026
Age	−0.026	0.017
Gender	−0.155	0.349
Practice order	−0.275	0.148

* Indicates *p* < 0.05;

** Indicates *p* < 0.01;

*** Indicates *p* < 0.005.

Given that gender was the only predictor that violates the PO assumption, we also ran multinomial logistic regression as a follow-up to ordinal logistic regression. Similarly, as shown in Table 4, the mindfulness composite was still significant for two categories: third preferred and least preferred. This suggested that people who have higher scores in these two facets of mindfulness tended to prefer OM more than other techniques and were more likely to rank it as their most preferred technique, rather than third preferred or least preferred. Additionally, perceived stress in our exploratory analysis again showed significance in the MNL model, such that higher perceived stress was related to greater likelihood of ranking OM as the most preferred technique, rather than third preferred or least preferred.

**Table 4:** Multinomial logistic regression of preferences for open monitoring.

	Second preferred		Third preferred		Least preferred	
Predictors	*Coefficient*	*SE*	*Coefficient*	*SE*	*Coefficient*	*SE*
Mindfulness composite	−0.359	0.205	−0.604^**^	0.222	−0.479^**^	0.214
Openness to experience	0.005	0.041	0.020	0.043	0.013	0.042
Age	0.035	0.030	0.052	0.031	0.043	0.032
Gender	0.367	0.571	−0.900	0.659	−0.642	0.594
Practice order	−0.227	0.239	−0.028	0.253	0.410	0.258

* Indicates *p* < 0.05;

** Indicates *p* < 0.01;

*** Indicates *p* < 0.005.

### Focused Attention

Attention focusing from ACS, acting with awareness from FFMQ, and conscientiousness from NEO-five factor inventory were used as predictors for FA in the ordinal logistic regression model. These predictors were highly correlated with each other (*p* < 0.001) – focusing and acting with awareness (*r* = 0.70), focusing and conscientiousness (*r* = 0.55), and acting with awareness and conscientiousness (*r* = 0.62). We ran separate models for each predictor, since given their conceptual distinctness, it was not deemed sensible to combine them into a composite. However, none of the predictors significantly predicted preferences for focused attention as shown in Table 5. Interestingly, gender was the only covariate that showed significance in the separate models, such that males were more likely to rank focused attention as the most preferred technique than females. For the primary predictors that showed null effects, it is potentially instructive to note the trends of predictors, although strong speculation is not warranted: attentional focusing and acting with awareness showed positive relationship in both the full and separate models, such that higher scores in attentional focusing and acting with awareness corresponded to greater probability of ranking focused attention as the most preferred technique versus all other categories; conversely, higher conscientiousness had a small negative coefficient in both full and separate models. Lastly, all predictors met the PO assumption, so multinomial logistic regression was not conducted. Our exploratory analysis with perceived stress and absorption as additional covariates did not change the significance of any of the variables, except that gender was non-significant.

**Table 5:** Ordinal logistic regression of preferences for focused attention.

	Full Model		Model 1		Model 2		Model 3	
Predictors	*Coefficient*	*SE*	*Coefficient*	*SE*	*Coefficient*	*SE*	*Coefficient*	*SE*
Attention focusing	0.427	0.337	0.244	0.236	-	-	-	-
Conscientiousness	−0.036	0.026	-	-	−0.015	0.020	-	-
Acting with awareness	0.044	0.305	-	-	-	-	0.079	0.196
Age	−0.001	0.017	−0.002	0.016	0.001	0.016	−0.002	0.016
Gender	−0.635	0.352	−0.699^*^	0.345	−0.694^*^	0.345	−0.684^*^	0.347
Practice order	−0.064	0.158	−0.046	0.150	−0.043	0.150	−0.057	0.155

* Indicates *p* < 0.05;

** Indicates *p* < 0.01;

*** Indicates *p* < 0.005.

### Body Scan

Attention shifting from ACS and sensory processing sensitivity as measured by the highly sensitive person scale (HSP) were included as predictors of preferences for body scan. Given that these two predictors were also highly correlated (*r* = −0.34, *p* < 0.001), separate regression models with each predictor were conducted. None of the predictors showed significance in predicting BS, which precludes strong interpretation. Nevertheless, people with higher attentional shifting capacity were less likely to rank body scan as their most preferred technique, whereas people with higher sensitivity to sensory stimuli and experiences were more likely to rank body scan as their most preferred technique. Lastly, none of the covariates showed significance in predicting BS. Table 6 shows estimates of coefficients and standard errors of all predictors. Given that the PO assumption was not violated by the predictors, multinomial logistic regression was not conducted. Our exploratory analysis also showed the same non-significance for all predictors.

**Table 6:** Ordinal logistic regression of preferences for body scan.

	Full Model		Model 1		Model 2	
Predictors	*Coefficient*	*SE*	*Coefficient*	*SE*	*Coefficient*	*SE*
Attention shifting	−0.158	0.257	−0.016	0.239	-	-
Sensory processing sensitivity	0.004	0.184	-	-	0.038	0.171
Age	0.026	0.018	0.026	0.018	0.024	0.017
Gender	−0.372	0.371	−0.369	0.345	−0.363	0.365
Practice order	−0.200	0.149	−0.200	0.149	−0.190	0.149

* Indicates *p* < 0.05;

** Indicates *p* < 0.01;

*** Indicates *p* < 0.005.

### Dissociable Preferences

**Figure 2:**
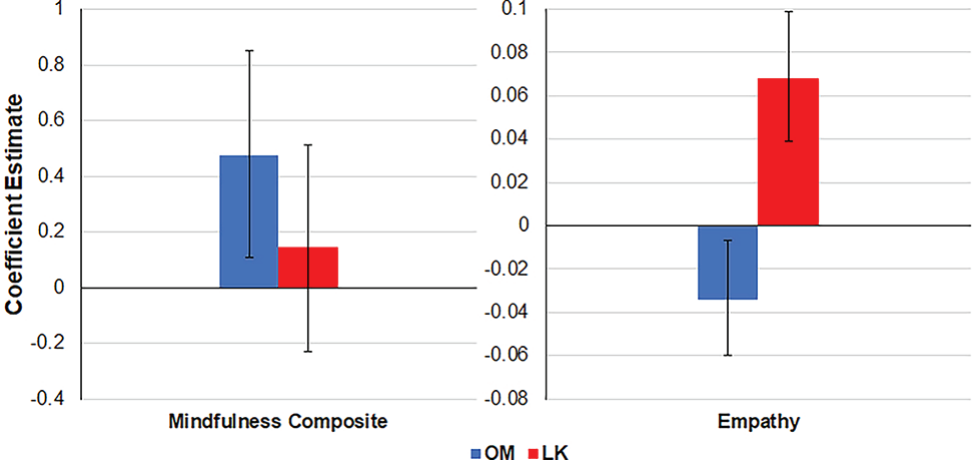
Dissociable preferences with primary predictors of OM and LK.

Based on the above results showing individual difference factors predicting preferences for open monitoring and loving-kindness, we conducted a second phase of analysis, that further tested the specificity of the relationship between the two mindfulness practices and the relevant individual difference factors. For this second phase of analysis, the trait mindfulness composite was added as a predictor of loving-kindness, whereas empathy and self-compassion were added as predictors of open monitoring. All other variables in the original models remained the same, including the covariates and other non-significant predictors. Results indicated that even after adding in predictors of the other technique, both loving-kindness and open monitoring still had their original predictors showing significance. Interestingly, empathy was found to be a negative yet significant predictor of OM after being added to the OM model. To further test the specificity of the empathy predictor, we conducted additional supplemental analyses including it as a predictor for FA and BS, but found no effects (see Supplementary Materials). These results demonstrate clear specificity in preferences for open monitoring and loving-kindness, since the predictive relationships with empathy and trait mindfulness were clearly dissociable. Figure 2 illustrates the selective relationships between the two individual difference factors and mindfulness practices, by indicating the coefficient estimates and 95% confidence interval of the primary predictors for each technique (note that the confidence intervals indicate which coefficients are reliably different from zero, but should not be used to compare across coefficients).

### Preferences and State Mindfulness

**Figure 3:**
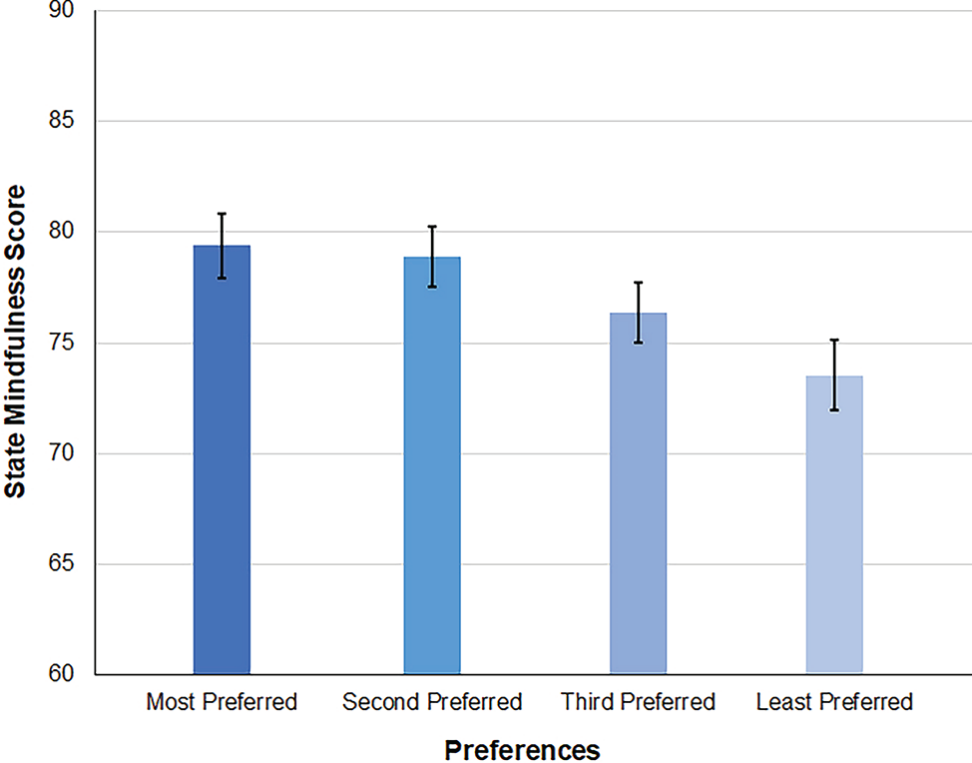
Mean of state mindfulness scores across preferences rankings.

To investigate if there is a correspondence between preference rankings and individuals’ evaluation of how well they were able to meditate with each of the techniques, we separately averaged the state mindfulness scores for each individual’s most preferred technique, second preferred technique, third preferred technique, and least preferred technique. A one-way ANOVA was conducted to examine mean differences in state mindfulness scores across the four different preference rankings. Results showed that there were significant mean differences in state mindfulness across the four different rankings (*F*(3, 472) = 3.45, *p* = 0.017). Furthermore, Tukey’s *post hoc* tests indicated that the state mindfulness scores of the most preferred technique (*M* = 79.39, *SD* = 15.74) was significantly higher than that of the least preferred technique (*M* = 73.54, *SD* = 17.33), and that the state mindfulness scores of the second preferred technique (*M* = 78.87, *SD* = 14.96) was also significantly higher than that of the least preferred technique (shown in Figure 3). Although no significant differences were detected between other rankings, the means of state mindfulness scores, follow a clear ordinal pattern, decreasing as the rankings go from most preferred to least preferred. This finding suggested that individuals had higher state mindfulness when they practiced their most and second preferred techniques relative to their least preferred technique.

## Discussion

The present study investigated whether individual preferences for four prototypical mindfulness techniques in MBIs can be predicted by personality traits or other dispositional markers. Additionally, the study also explored whether preferences for different techniques have meaningful implications for how well individuals are able to enter a meditative state. The results indicated that preferences for two of the techniques – loving-kindness and open monitoring – were predicted by related traits; yet conversely, none of the hypothesized traits were significant predictors for focused attention or body scan. Nonetheless, there was a positive correspondence between preference rankings and level of state mindfulness, supporting the hypothesis that individual preferences could have an impact on meditative state.

Consistent with our hypothesis, preferences for loving-kindness were significantly predicted by empathy and agreeableness, when examined separately. Comparing empathy and agreeableness, the fit indices favored the model with empathy. Moreover, empathy remained to be a significant predictor even after controlling for the effect of agreeableness. This is not surprising because successfully cultivating loving-kindness toward others would rely on engaging empathic qualities such as perspective taking (i.e., putting oneself in other’s shoes) and empathic concerns for others’ suffering (Luberto et al., 2018). Relatedly, loving-kindness has been shown to improve empathy and related constructs such as prosocial behavior and compassion (Singer and Klimecki, 2014), which further suggests that empathy is an important ingredient in the practice of loving-kindness meditation. In contrast, agreeableness includes characteristics such as cooperativeness and adherence, which are qualities not explicitly involved in fostering loving-kindness. These may explain why agreeableness was a less robust predictor.

However, self-compassion showed an opposite, albeit less reliable trend, which was unexpected given that self-compassion should also facilitate loving-kindness practice, especially in the practice of generating kindness toward oneself. This perplexing result may be due to the fact that not all components of self-compassion scale are related to loving-kindness practice (i.e., it may be useful to examine subscales of self-compassion more closely, to determine whether those related to practicing loving-kindness are positive predictors). However, in this study we used the total score of self-compassion rather than individual subscales, as we did not have any *a priori* hypotheses relating to subscales of self-compassion. Instead, we examined overall self-compassion capacity with respect to loving-kindness preferences. Finally, gender was a significant predictor of loving kindness, such that females preferred loving kindness more so than males. This sex differences in preferences were not part of our original hypotheses but were not surprising based on previous literature that showed reliable sex differences in empathy. Specifically, females in general do appear to be more empathic than males and exhibit higher emotional responsiveness in affective empathy than males (Christov-Moore et al., 2014), which may explain why females exhibited more preferences for a technique that involves empathic qualities.

Similarly, preferences for open monitoring were predicted by traits that have close associations with the actual practice (e.g., nonjudgment of internal experiences and nonreactivity to internal experiences). As described in introduction, being nonjudgmental and non-reactive to internal and external experiences are key components emphasized in open monitoring meditation. Therefore, individuals having high trait mindfulness in these two facets may experience a shallower learning curve when first exposed to open monitoring practices, thereby leading to stronger preferences for this particular technique. However, openness to experience did not significantly predict open monitoring as we expected. Additionally, it was surprising to see that in our exploratory analysis, perceived stress was a covariate that showed significance in predicting preferences for open monitoring. Although it is unclear as to why a high level of perceived stress would predict more preferences for this technique, one plausible explanation may be that the emphasis of nonjudgment and nonreactivity toward present moment experiences in open monitoring practice, including stressful thoughts, may actually serve to reduce stress to some extent, leading to a stronger preference for this type of practice. In fact, one recent study did show that brief mindfulness-based monitoring and acceptance training can induce reduction in physiological measures of stress reactivity (Lindsay et al., 2018). Nonetheless, our result suggests that perceived stress is worth investigating further as a primary predictor in future studies, since it did show specificity in predicting preferences for open monitoring, but not the other three techniques.

Inconsistent with our hypotheses, focused attention and body scan were not significantly predicted by any of the hypothesized predictors in the present study. However, for focused attention, all predictors except conscientiousness, were in the expected directions, such that higher capacities in attentional focusing and acting with awareness was associated with a tendency to prefer this technique. Additionally, in follow-up analyses, we detected significance of gender in predicting preferences for FA, even though this factor did not reach significance in the primary analysis. It is unclear as to why males were more likely to rank FA as their most preferred technique than females. More targeted studies focusing on gender differences in preferences for mindfulness techniques would be needed to fully validate this preliminary finding. For the body scan, higher sensory processing sensitivity and lower attentional shifting were weakly related to preferences for body scan, which was not supportive of our theoretical hypotheses.

These nonsignificant findings of hypothesized traits associated with FA and BS preferences suggest two possible explanations. First, there could be other more sensitive and optimized predictors for these two mindfulness techniques that were not included in the present study. Relatedly, it is also possible that the self-report instruments used in this study did not adequately capture the traits or characteristics that were being evaluated. For instance, unlike conventional personality traits, individuals’ self-reported or subjective perception of their capacities and abilities, such as attentional focusing and attentional shifting, may be prone to various reporting biases (Furnham and Ward, 2001; Dunning et al., 2004). In contrast, objective cognitive tasks of attentional control may be able to more accurately and reliably assess these attentional abilities. Second, despite our hypotheses postulating that preferences for each technique should be predicted by individual variability in certain traits, it is still possible that there may be no meaningful variation to be predicted for these two techniques (FA and BS). In other words, focused attention and body scan may not be associated with systematic preferences that could be linked to trait characteristics. However, both explanations necessitate validation from future investigations that explore other promising predictors and sources of individual variability, given that the present study was only a first attempt to predict individual preferences for mindfulness techniques from personality and dispositional traits.

Finally, for the two techniques predicted by personality and other dispositional traits – loving-kindness and open monitoring – we were able to further show the specificity effect of each predictor, in that each was associated with the theoretically-linked technique even after controlling for the other predictor. This finding suggests that the predictive power of individual trait characteristics was not due to chance and that robustly dissociable preference profiles can be detected for these two techniques. Furthermore, we also demonstrated that individuals had higher state mindfulness when practicing their preferred techniques, relative to their less preferred techniques, illustrating the practical implications of assessing and predicting individual preferences. Notably, this finding speaks to the critical role of individual preferences in affecting how well individuals can engage with a practice and enter into a meditative state. Previous studies have shown that improvement in state mindfulness over the course of MBIs is related to improvement in psychological outcomes at post-intervention (Gayner et al., 2012; Kiken et al., 2015). Therefore, it is possible that assigning individuals to their preferred technique would likely increase their state mindfulness over the course of intervention, thereby resulting in greater improvement in targeted outcomes at the end of intervention.

### Limitations and Future Directions

The present study does have several limitations that warrant further replication and investigations. First, we did not examine all putative predictors for each mindfulness technique, thus it is highly plausible that we missed meaningful variables that could otherwise be predictive of individual preferences. Given that this is the first study exploring this research question, it was not feasible to include a large set of different dispositional traits or other relevant individual characteristics in this investigation. Moreover, this would have reduced statistical power, by opening up the analysis to increased multiple comparisons and related concerns (i.e., those related to “fishing expeditions”). Nevertheless, additional studies are needed to examine other potential predictors of mindfulness preferences to determine whether there are potentially more sensitive sources of individual variability. Second, all traits were assessed based on self-report questionnaires in the present study, which could be prone to potential subjective biases, especially with regard to cognitive related abilities, such as attentional control. Future investigations would benefit from employing cognitive tasks and other objective measures of putative individual traits and characteristics. Third, we did not examine all potentially meaningful types of mindfulness practices (e.g., Mantra meditation or Zen styles), in the present study due to concern of having multiple sessions spanning over for more than a week, which could pose challenges for participant retention. Future studies may want to explore other useful techniques and their relevant predictors based on the current findings. Fourth, our study was conducted online with multiple quality control steps built in place; nevertheless, it is possible that in-person instructions of mindfulness techniques may be different from listening to pre-recorded audio-guided instructions by certified mindfulness instructor. Further replication of the present study with face-to-face mindfulness training or even online live-stream guided instructions by certified instructor would be necessary to validate these results, as these delivery formats may potentially increase the engagement of participants. Lastly, an interesting future direction worth exploring is whether preferences for mindfulness techniques are fixed or malleable over the course of MBIs. In particular, the current study focused solely on individuals that were naïve to mindfulness and did not track whether preferences changed upon further practice and exposure. Indeed, it is quite possible that preferences are dynamic and changeable with practice. If such dynamic patterns can be observed, it would point to the need for understanding what factors can drive changes in preference. Such studies could be highly informative for intervention development and teaching, as it would enable better design and application of MBIs to increase individuals’ preferences and outcomes.

## Conclusion

Understanding individual differences in response to standardized MBIs is critical for improving intervention effectiveness and outcomes. The present study provided a first step in tackling this individual difference question, by examining individual preferences for different mindfulness techniques commonly taught in MBIs. The results illustrate that individuals not only have different preferences for individual mindfulness technique, which can be predicted by personality and dispositional traits, but also are more likely to prefer techniques compatible with their personality or dispositional traits. These findings provide support for personalizing MBIs to better meet individual needs, while also highlighting the need for future scientific investigations and clinical applications that consider these individual differences with respect to treatment planning and outcome evaluation.

## Data Availability Statement

The raw data supporting the conclusions of this article will be made available by the authors, without undue reservation, to any qualified researcher.

## Ethics Statement

The studies involving human participants were reviewed and approved by Institutional Review Board at Washington University in St. Louis. The patients/participants provided their written informed consent to participate in this study.

## Author Contributions

RT contributed to study design, data collection, writing and critical revision of the article, and final approval of the version to be published. TB contributed to study design, writing and critical revision of the article, and final approval of the version to be published.

## Conflict of Interest

The authors declare that the research was conducted in the absence of any commercial or financial relationships that could be construed as a potential conflict of interest.

## Supplementary Material

The Supplementary Material for this article can be found online at: https://www.frontiersin.org/articles/10.3389/fpsyg.2020.01163/full#supplementary-material.
