# Supplementary material for: Predicting Individual Preferences in Mindfulness Techniques Using Personality Traits
Source: Front Psychol. 2020 Jun 18;11:1163. doi: 10.3389/fpsyg.2020.01163 (PMC7314956; doi:10.3389/fpsyg.2020.01163)
Supplement: Supplementary file 1 [file Data_Sheet_1.docx]

**Supplementary Materials**

**Results including perceived stress and absorption as covariates:**

Table 1. Ordinal Logistic Regression of Preferences for Loving-kindness.

|  | **Model 1** | | | | **Model 2** | | | |  |
| --- | --- | --- | --- | --- | --- | --- | --- | --- | --- |
| **Predictors** | *Coefficient* | | *SE* | | *Coefficient* | | *SE* | |  |
| Empathy | 0.060*** | 0.016 | | | - | - | | |  |
| Agreeableness | - | - | | | 0.061* | 0.028 | | |  |
| Self-Compassion | -0.284 | 0.298 | | | -0.321 | 0.298 | | |  |
| Age | -0.029 | 0.020 | | | -0.023 | 0.019 | | |  |
| Gender | 0.949* | 0.402 | | | 1.089** | 0.397 | | |  |
| Practice Order | -0.349* | 0.172 | | | -0.260 | 0.167 | | |  |
| Perceived Stress | -0.020 | 0.032 | | | -0.013 | 0.032 | | |  |
| Absorption | 0.036 | 0.028 | | | 0.078*** | 0.026 | | |  |
| **Model Fit**  AIC | 279.61 | | | 288.21 | | | |  |  |
| ^*^ Indicates p<0.05, ^**^ indicates p<0.01, and ^***^ indicates p<0.005. | | | | | | | | | |

Table 2. Multinomial Logistic Regression of Preferences for Loving-kindness.

|  | **2^nd^ Preferred** | | | **3^rd^ Preferred** | | | **Least Preferred** | | |
| --- | --- | --- | --- | --- | --- | --- | --- | --- | --- |
| **Predictors** | *Coefficient* | | *SE* | *Coefficient* | | *SE* | *Coefficient* | | *SE* |
| Empathy | -0.099*** | 0.032 | | -0.110*** | 0.034 | | -0.131*** | 0.032 | |
| Self-Compassion | 1.179* | 0.510 | | 1.284* | 0.563 | | 0.756 | 0.483 | |
| Age | -0.013 | 0.032 | | 0.008 | 0.035 | | 0.033 | 0.030 | |
| Gender | -0.882 | 0.630 | | -1.013 | 0.718 | | -1.771** | 0.638 | |
| Practice Order | 0.087 | 0.282 | | -0.053 | 0.326 | | 0.613* | 0.279 | |
| Perceived Stress | 0.038 | 0.052 | | 0.044 | 0.058 | | 0.045 | 0.049 | |
| Absorption | -0.009 | 0.045 | | -0.021 | 0.051 | | -0.048 | 0.045 | |
| ^*^ Indicates p<0.05, ^**^ indicates p<0.01, and ^***^ indicates p<0.005. | | | | | | | | | |

Table 3. Ordinal Logistic Regression of Preferences for Open Monitoring.

| **Predictors** | Coefficient | SE |
| --- | --- | --- |
| Mindfulness Composite | 0.578*** | 0.168 |
| Openness to Experience | 0.013 | 0.030 |
| Age | -0.033 | 0.018 |
| Gender | -0.173 | 0.372 |
| Practice Order | -0.290 | 0.156 |
| Perceived Stress | 0.058* | 0.026 |
| Absorption | -0.052 | 0.027 |
| ^*^ Indicates p<0.05, ^**^ indicates p<0.01, and ^***^ indicates p<0.005. | | |

Table 4. Multinomial Logistic Regression of Preferences for Open Monitoring.

|  | **2^nd^ Preferred** | | | **3^rd^ Preferred** | | | **Least Preferred** | | |
| --- | --- | --- | --- | --- | --- | --- | --- | --- | --- |
| **Predictors** | *Coefficient* | | *SE* | *Coefficient* | | *SE* | *Coefficient* | | *SE* |
| Mindfulness Composite | -0.595* | 0.299 | | -1.011*** | 0.318 | | -0.967*** | 0.326 | |
| Openness to Experience | -0.053 | 0.052 | | -0.029 | 0.054 | | -0.035 | 0.055 | |
| Age | 0.025 | 0.032 | | 0.047 | 0.033 | | 0.045 | 0.035 | |
| Gender | 0.168 | 0.612 | | -0.905 | 0.696 | | 0.739 | 0.657 | |
| Practice Order | -0.204 | 0.250 | | 0.003 | 0.269 | | 0.444 | 0.290 | |
| Perceived Stress | -0.065 | 0.047 | | -0.101* | 0.049 | | -0.124* | 0.052 | |
| Absorption | 0.092* | 0.047 | | 0.075 | 0.049 | | 0.110* | 0.050 | |
| ^*^ Indicates p<0.05, ^**^ indicates p<0.01, and ^***^ indicates p<0.005. | | | | | | | | | |

Table 5. Ordinal Logistic Regression of Preferences for Focused Attention.

|  | **Full Model** | | | **Model 1** | | | **Model 2** | | | **Model 3** | | |
| --- | --- | --- | --- | --- | --- | --- | --- | --- | --- | --- | --- | --- |
| **Predictors** | *Coefficient* | | *SE* | *Coefficient* | | *SE* | *Coefficient* | | *SE* | *Coefficient* | | *SE* |
| Attention Focusing | 0.452 | 0.347 | | 0.264 | 0.269 | | - | - | | - | - | |
| Conscientiousness | -0.028 | 0.028 | | - | - | | -0.017 | 0.023 | | - | - | |
| Acting with Awareness | -0.073 | 0.318 | | - | - | | - | - | | 0.013 | 0.224 | |
| Age | 0.002 | 0.018 | | -0.001 | 0.017 | | 0.002 | 0.017 | | 0.000 | 0.017 | |
| Gender | -0.632 | 0.373 | | -0.673 | 0.366 | | -0.630 | 0.367 | | -0.661 | 0.365 | |
| Practice Order | -0.013 | 0.165 | | -0.007 | 0.157 | | -0.012 | 0.158 | | -0.006 | 0.160 | |
| Perceived Stress | -0.003 | 0.022 | | 0.003 | 0.021 | | -0.012 | 0.021 | | -0.005 | 0.021 | |
| Absorption | -0.020 | 0.023 | | -0.027 | 0.022 | | -0.021 | 0.023 | | -0.025 | 0.022 | |
| ^*^ Indicates p<0.05, ^**^ indicates p<0.01, and ^***^ indicates p<0.005. | | | | | | | | | | | | |

Table 6. Ordinal Logistic Regression of Preferences for Body Scan.

|  | **Full Model** | | | **Model 1** | | | **Model 2** | | |
| --- | --- | --- | --- | --- | --- | --- | --- | --- | --- |
| **Predictors** | *Coefficient* | | *SE* | *Coefficient* | | *SE* | *Coefficient* | | *SE* |
| Attention Shifting | -0.045 | 0.315 | | -0.017 | 0.307 | | - | - | |
| Sensory Processing Sensitivity | -0.095 | 0.241 | | - | - | | -0.088 | 0.235 | |
| Age | 0.035 | 0.018 | | 0.035 | 0.018 | | 0.035 | 0.018 | |
| Gender | -0.296 | 0.382 | | -0.337 | 0.368 | | -0.306 | 0.376 | |
| Practice Order | -0.220 | 0.153 | | -0.222 | 0.153 | | -0.220 | 0.153 | |
| Perceived Stress | 0.021 | 0.027 | | 0.016 | 0.024 | | 0.023 | 0.025 | |
| Absorption | 0.005 | 0.025 | | 0.000 | 0.022 | | 0.004 | 0.024 | |
| ^*^ Indicates p<0.05, ^**^ indicates p<0.01, and ^***^ indicates p<0.005. | | | | | | | | | |

**Dissociable Preferences (with absorption and perceived stress added as additional covariates):**

Dissociable Preferences with Primary Predictors of OM and LK.

|  | **OM** | | | **LK** | | |  |
| --- | --- | --- | --- | --- | --- | --- | --- |
| **Predictors** | *Coefficient* | | *SE* | *Coefficient* | | *SE* |  |
| Empathy | -0.026 | 0.014 | | 0.062*** | 0.016 | |  |
| Self-Compassion | 0.203 | 0.306 | | -0.429 | 0.339 | |  |
| Mindfulness Composite | 0.524** | 0.196 | | 0.180 | 0.200 | |  |
| Gender | -0.158 | 0.379 | | 0.969* | 0.402 | |  |
| ^*^ Indicates p<0.05, ^**^ indicates p<0.01, and ^***^ indicates p<0.005. | | | | | | | |

**Empathy Predicting FA and BS (including age, gender, and practice order as covariates):**

|  | **FA** | | | **BS** | | |  |
| --- | --- | --- | --- | --- | --- | --- | --- |
| **Predictors** | *Coefficient* | | *SE* | *Coefficient* | | *SE* |  |
| Empathy | -0.010 | 0.011 | | -0.018 | 0.012 | |  |
| ^*^ Indicates p<0.05, ^**^ indicates p<0.01, and ^***^ indicates p<0.005. | | | | | | | |

**Demographics and Personality Comparison for Completed and Drop-out Participants:**

We first examined the demographics (i.e. age and gender) between completed and drop-out participants. While the range of age was equivalent between the two groups, the drop-out group had people who were significantly younger (*M*=32.8, *SD*=9.11) than the completed group (*M*= 36.9, *SD*= 10.0). However, two groups were not statistically different in gender distribution.

We then compared group differences in the Big Five personality factors, as well as predictor variables that showed significance in our main analyses: empathy and trait mindfulness composite. It should be noted that majority of these people dropped after the first session (questionnaires) and before the first mindfulness session. Their answers to questionnaires may not be reliable because of missing answers and random filings on many items, thus statistical differences in these personality and predictor variables between the two group should be treated with caution.

While no significant group difference was observed for neuroticism, people in the completed group had significantly lower extraversion, but higher openness, agreeableness, and conscientiousness, than the drop-out group.

|  | **Group** | **Mean** | **SD** |
| --- | --- | --- | --- |
| Neuroticism | Completed | 32.53 | 12.23 |
|  | Drop-out | 34.48 | 10.29 |
| Extraversion* | Completed | 34.35 | 9.94 |
|  | Drop-out | 37.47 | 8.55 |
| Openness*** | Completed | 41.72 | 6.86 |
|  | Drop-out | 38.00 | 6.80 |
| Agreeableness** | Completed | 43.80 | 7.88 |
|  | Drop-out | 40.41 | 9.69 |
| Conscientiousness** | Completed | 46.60 | 8.41 |
|  | Drop-out | 43.12 | 9.01 |
| **Indicated p< 0.05, ** indicates p<0.01, and *** indicates p<0.005* | | | |

For our primary predictor variables (empathy and trait mindfulness composite), no significant difference was detected between the two groups.
